# Structural properties and electromagnetic shielding performance of bidirectional carbon fabric and Fe_2_O_3_ nano filler reinforced epoxy composites

**DOI:** 10.1039/d5ra06245d

**Published:** 2025-10-22

**Authors:** R. Suresha, H. K. Sachidananda, B. Shivamurthy, Gibin George, Sampath Parasuram

**Affiliations:** a School of Engineering & Information Technology, Department of Electrical & Electronics Engineering, Manipal Academy of Higher Education Dubai United Arab Emirates suresha@manipaldubai.com; b School of Engineering & Information Technology, Department of Mechanical Engineering, Manipal Academy of Higher Education Dubai United Arab Emirates sachidananda@manipaldubai.com; c Department of Mechanical & Industrial Engineering, Manipal Institute of Technology, Manipal Academy of Higher Education Manipal-576104 India shiva.b@manipal.edu; d Department of Mechanical Engineering, SCMS School of Engineering and Technology Karukutty Kerala India gibingeorge@scmsgroup.org; e Department of Materials Engineering, Indian Institute of Science Bangalore India parasurams@iisc.ac.in

## Abstract

This study investigates the structural and electromagnetic interference (EMI) shielding performance of bidirectional carbon fabric/epoxy composites reinforced with Fe_2_O_3_ nanoparticles for aerospace applications. Composites with varying Fe_2_O_3_ loadings (1–3 wt%) were fabricated using a hand lay-up method, followed by mechanical and electromagnetic shielding characterisation in the X-band frequency range (8.2–12.4 GHz). Tensile testing revealed that 1–2 wt% Fe_2_O_3_ loading enhanced the stiffness and tensile strength of the composite due to improved fibre–matrix interfacial bonding, while 3 wt% caused agglomeration and reduced strength. EMI shielding measurements showed absorption-dominated performance across all configurations, with multiple layers significantly improving total shielding effectiveness (SE_T_). The highest SE_T_ (25.3 dB) was achieved for a two-layer laminate with 3 wt% Fe_2_O_3_, attributed to synergistic dielectric and magnetic losses and enhanced internal reflections. The results demonstrate that optimised Fe_2_O_3_ content and laminate layering can deliver lightweight, structurally robust composites with effective EMI shielding, making them suitable for advanced aerospace structures requiring mechanical integrity and electromagnetic compatibility. These findings highlight that optimized nanoparticle loading, and laminate architecture can yield lightweight composites that unite mechanical robustness with effective EMI shielding, offering strong potential for aerospace structures demanding both structural performance and electromagnetic compatibility.

## Abstract 2

This study investigates the structural and electromagnetic interference (EMI) shielding performance of bidirectional carbon fabric/epoxy composites reinforced with Fe_2_O_3_ nanoparticles for aerospace applications.

## Graphical abstract

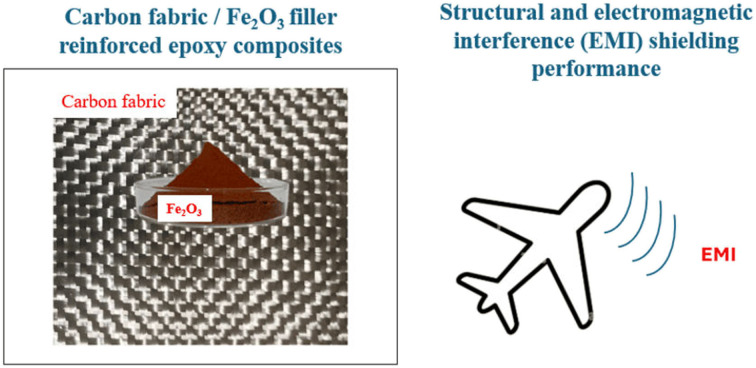

## 1. Introduction

Modern avionics and onboard systems integrate dense digital avionics, networked sensors, and wireless links, heightening susceptibility to electromagnetic interference from both onboard systems and passenger devices. Uncontrolled EMI degrades signal integrity and can trigger malfunctions or failures in safety-critical functions, undermining operational reliability and flight safety.^1^

In addition to onboard sources, aircraft encounter external EMI from thunderstorms, electrostatic discharges, and solar activity, making shielding essential for flight safety.^2^ At the same time, the drive for lightweight, high-performance structures has accelerated the use of carbon fibre-reinforced epoxy (CF/E) composites, which combine high specific strength, fatigue resistance, thermal stability, and corrosion resistance, positioning them as attractive alternatives to traditional metals in aerospace applications.^3^

Although carbon fiber/epoxy composites provide excellent structural performance, their semiconducting nature limits intrinsic EMI shielding. Consequently, research increasingly targets enhancing their effectiveness in the 8–12 GHz X-band frequency, a range vital for radar, satellite communication, navigation, and wireless networks, where reliable EMI protection is essential for uninterrupted operation.^4^

Bidirectional carbon fabric/epoxy composites offer superior EMI shielding compared to unidirectional ones, but excessive nanofiller addition can cause agglomeration and degrade mechanical strength, underscoring the need for optimized loading.^5^ Recent studies emphasize strategies such as incorporating magnetic nanofillers, forming conductive networks, and designing multilayer structures to enhance shielding effectiveness in the X-band (8.2–12.4 GHz).

Ahmad *et al.*^6^ showed that alternating carbon fiber plies reinforced with MWCNTs and Fe_2_O_3_ enhanced EMI shielding *via* combined dielectric and magnetic losses. Waseem *et al.*^7^ developed a hybrid layered composite using ZrB_2_-SiC, Fe_3_O_4_-loaded carbon fabric, and carbonized cotton fibre, achieving lightweight, thermally stable, absorption-dominated shielding. Shao *et al.*^8^ reported a Janus-structured CNT/Fe_3_O_4_/MXene membrane (∼84.9 μm) with 44.56 dB shielding in the X-band, along with excellent thermal conductivity, flexibility, and low weight. Likewise, Yu *et al.*^9^ created a nacre-inspired Fe_3_O_4_/CNT composite with alternating conductive and magnetic layers, delivering durable, absorption-dominant shielding that retained 96% efficiency after physical damage.

Zhang *et al.*^10^ engineered ultralight rGO–CNT–epoxy aerogels with Fe_3_O_4_ nanoparticles, achieving a reflection loss of −58.13 dB at 12.08 GHz through a 3D porous structure that enhanced scattering and impedance matching. Wilson *et al.*^11^ reported TPU laminates with graphite and CoFe_2_O_4_, where the G/F/G configuration reached approximately 54 dB shielding from synergistic dielectric and magnetic losses. Durmaz *et al.*^12^ developed bio-based PA11/PLA composites with 30 wt% carbon fibres, yielding 28 dB SE at 10 GHz through reflection-dominated shielding. Ahmad *et al.*^13^ demonstrated graphene-Fe_2_O_3_ polymer composites with broadband EMI attenuation from combined dielectric reflection and magnetic absorption. Duan *et al.*^14^ fabricated CF/GO/Fe_3_O_4_/epoxy multilayers that improved impedance matching and promoted absorption-based shielding. Fallah *et al.*^15^ achieved 36.6 dB SE at 8.2 GHz in epoxy nanocomposites with Fe_2_O_3_ and carbon black, showing tunable frequency response. Bhaskaran *et al.*^16^ developed Fe_3_O_4_@GNP hybrids with ∼9.6 dB SE at 1 mm, outperforming individual fillers through synergistic effects. Xu *et al.*^17^ produced epoxy-cotton fibre scaffolds with GNPs and Fe_3_O_4_, delivering 33.1 dB SE_T_ and absorption-dominant shielding with structural stability for defence and electronics.

Veeramani *et al.*^18^ studied carbon fiber/epoxy composites fabricated *via* vacuum-assisted resin infusion, incorporating polyphenylene ether (PPE) as a toughening agent. PPE dispersed in the interlaminar region enhanced toughness, with DMA results showing a notable increase in glass transition temperature. In another work, Veeramani *et al.*^19^ developed self-healing composites by embedding urea-formaldehyde-encapsulated dicyclopentadiene (DCPD) microcapsules with epoxy, chopped CF, and CNTs. These systems enabled self-detection and prevention of microcrack propagation, with potential applications in aerospace, wind energy, and marine structures. Eken *et al.*^20^ investigated two-layer CFRP composites reinforced with hematite (Fe_2_O_3_) and goethite (FeO(OH)) produced by hand lay-up, evaluating EMI shielding across 700–6000 MHz and impact resistance. They reported overall improvements in both shielding effectiveness and impact strength compared to unreinforced samples.

Fe_2_O_3_ was selected in this study due to its lower cost, environmental benignity, chemical stability, and strong magnetic loss response in the X-band compared to fillers like Fe_3_O_4_ and CoFe_2_O_4_. Unlike earlier works that broadly combined carbon-based conductive and magnetic fillers, the novelty of this research lies in optimizing Fe_2_O_3_ loading within carbon fabric/epoxy laminates to simultaneously balance structural performance and absorption-dominated EMI shielding. The exclusive focus on the X-band is justified by its critical importance in aerospace applications, particularly radar, satellite communication, and navigation systems, where reliable EMI protection is essential for operational safety.

Previous studies on conductive and magnetic nanofillers have advanced EMI shielding but often overlooked structural robustness. Next-generation aerospace, defence, and wearable systems require materials that are both lightweight and multifunctional. This study addresses that gap by investigating Fe_2_O_3_-reinforced carbon fabric/epoxy composites, linking shielding effectiveness with mechanical performance. By integrating structural strength with absorption-dominated shielding, the work proposes a multifunctional composite system that meets aerospace demands for both mechanical integrity and electromagnetic compatibility, thereby contributing to the design of advanced materials capable of balancing performance requirements often neglected in earlier research.

## 2. Materials and methods

In this research work, the epoxy matrix is reinforced with bidirectional carbon fabric and Fe_2_O_3_ nanoparticles to prepare composites.

### 2.1. Materials

**Fig. 1:**
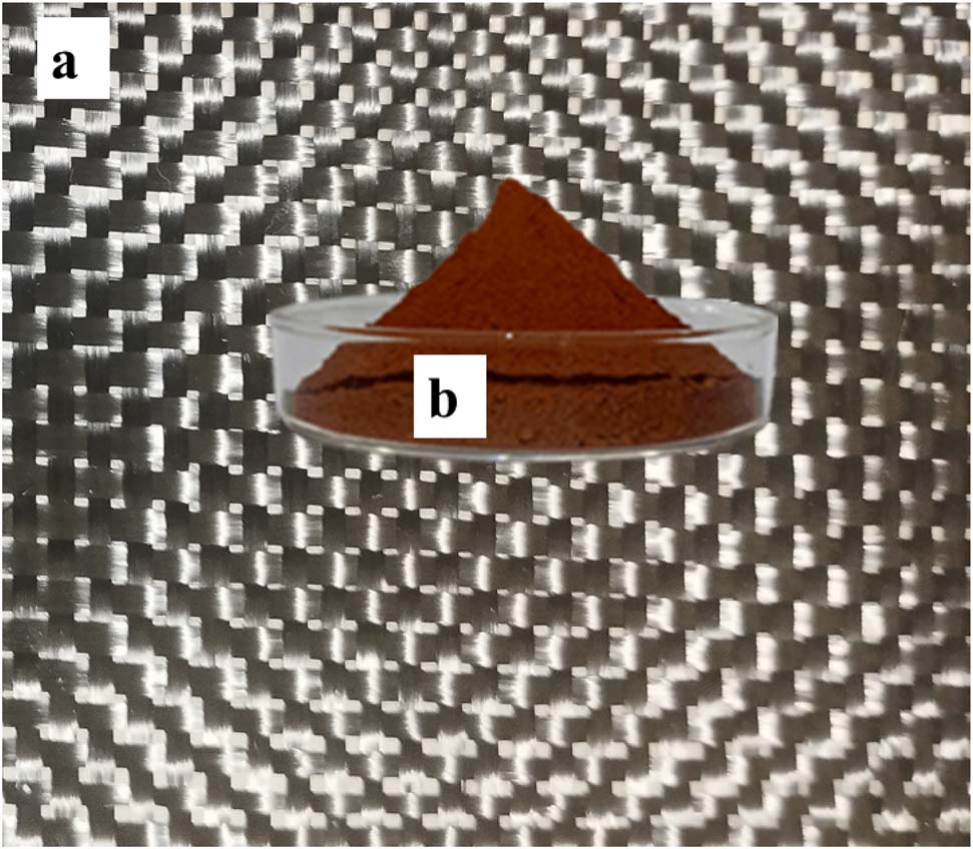
Digital photographs of (a) carbon fabric, (b) Fe_2_O_3_.

The bi-directional oriented plain weave type carbon fabric supplied by M/s Bhor Chemical & Plastics Pvt. Ltd, Nasik, India, is used as primary reinforcement. The material batch number: W21A31/3298, the area density of the carbon fabric used, and the type of fibre were 160 g m^−2^ ± 5%, and 3k, respectively. Fig. 1(a) is the digital photograph of the carbon fabric used for this study. Bisphenol-A epoxy, combined with a suitable hardener supplied by M/s Addnano, was used as matrix material. Fe_2_O_3_ Nanofillers (Fig. 1(b)) are procured from M/s Addnano, used as secondary reinforcement. The density of the oxide is about 5.3 g cm^−3^, molecular weight 159.6 g mol^−1^, and melting point about 1565 °C. All these components are used as received for processing the composite. In this study, we used diglycidyl ether of bisphenol-A (DGEBA, LY 556 grade, Huntsman) as the epoxy resin, cured with aromatic amine hardener (HY 951) at a 100 : 20 resin-to-hardener weight ratio, following an amine addition curing mechanism. Ethanol was chosen as the dispersant for Fe_2_O_3_ nanoparticles due to its low toxicity, high volatility, and good compatibility with both the epoxy system and nanoparticle surfaces, which facilitated uniform dispersion while minimizing health and environmental hazards compared to DMF or acetone. To ensure complete removal of residual ethanol prior to curing, the nanoparticle–resin mixtures were subjected to continuous magnetic stirring and mild heating at 60 °C, followed by vacuum drying, thereby eliminating solvent traces that could otherwise affect curing or composite integrity.

### 2.2. Preparation of the Fe_2_O_3_ dispersed epoxy matrix

When preparing Fe_2_O_3_ nanoparticle-loaded carbon fibre-reinforced epoxy composites, it is paramount that the nanofillers are evenly dispersed within the epoxy matrix. Nanofiller dispersion in the matrix allows for an efficient loading of the reinforced area, which can positively affect load transfer from the matrix to the reinforcements. This eventual loading establishes and extends the interfaces between the nanofillers and polymer matrix, leading to improved strength of the matrix, in addition to other functional attributes. However, high viscosity and low solubility of the epoxy polymer are significant hurdles to effective nanofiller dispersion in the polymer matrix. Solvents such as tetrahydrofuran (THF), dimethylformamide (DMF), acetone, ethanol, water, dichloromethane (DCM), and methyl ethyl ketone (MEK) have been utilised as dispersants to allow the nanofiller to be dispersed into the epoxy matrix. The most common methods used for dispersion have used techniques including tip sonication, bath sonication, mechanical mixing, shear mixing, and three-roll calendaring. Most of these methods of dispersion have been successful. In this study, ethanol was used as the solvent to disperse the Fe_2_O_3_ nanoparticles in the epoxy resin. Incorporating Fe_2_O_3_ nanoparticles into multifunctional epoxies like TGDDM or TGPAP would likely enhance EMI shielding stability at high temperatures, strengthen interfacial polarization effects, and improve mechanical/thermal endurance compared to DGEBA-based systems. The only challenges are related to brittleness and nanoparticle dispersion. These multifunctional epoxies provide a more robust and reliable platform for advanced EMI shielding composites, particularly for aerospace and defence applications where performance under extreme conditions is required.

**Fig. 2:**
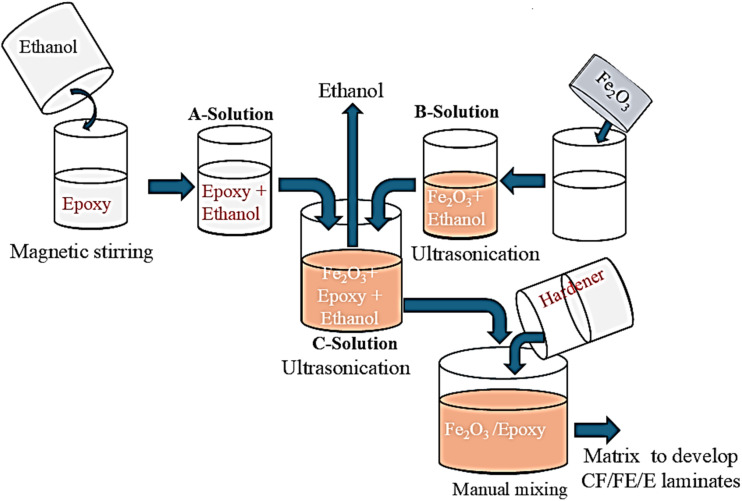
Fe_2_O_3_-filled epoxy matrix preparation.

The preparation of the Fe_2_O_3_-filled epoxy resin is illustrated in Fig. 2. First, equal amounts of ethanol and epoxy were mixed with a magnetic stirrer for 30 minutes and termed as solution A. In a separate container, Fe_2_O_3_ nanoparticle was dispersed in ethanol *via* a probe-type ultrasonicator for 2 hours, and this mixture was termed solution B. After a uniform dispersion of Fe_2_O_3_ in ethanol was achieved, solution B was mixed with solution A and ultrasonic dispersion was performed for an additional 2 hours. This mixture was termed solution C. Solution C was mixed with hardener and mechanically stirred for 5 minutes to prepare the hybrid matrix for composite laminate fabrication. This procedure for matrix preparation was repeated for different Fe_2_O_3_ nanoparticle concentrations of 1 wt%, 2 wt%, and 3 wt%. The material compositions of the hybrid matrix prepared using the previously described method are summarised in Table 1.

**Table 1:** Material constituents of the Fe_2_O_3_-filled epoxy resin

Sl no.	Sample designation	Fe_2_O_3_ (wt%)	Epoxy (vol%)	Hardener (vol%)
1	E	0	100	20
2	1FE/E	1	100	20
3	2FE/E	2	100	20
4	3FE/E	3	100	20

### 2.3. Fabrication of composite laminates

The bi-directional plain weave carbon fabric that the manufacturer provided was cut into 100 mm × 100 mm squares and immersed in acetone to remove sizing agents, dirt, contaminations, amorphous carbon, and grease. After soaking in the acetone, the mass was rinsed with deionized water and then placed in the hot air oven for 10 minutes to remove residual moisture.

The cleaned carbon fabric was then placed on a glass plate, and the prepared hybrid epoxy matrix was spread over the surface of the carbon fabric using a roller and brush. Similarly, fabrics were stacked one above the other, with the matrix applied between the fabric layers and then consolidated by pressing with a roller. Before placing the carbon fabric on the glass plate, a releasing film was used to facilitate easy removal of the laminate. Likewise, after completing the layup, a releasing film was placed on the top along with the glass plate. The entire stack, impregnated with resin, was kept at room temperature for curing, which took about 24 hours, resulting in the production of the laminates. Table 2 presents the material composition and designation of the composite sample laminates.

**Table 2:** Constituents and designations of the prepared composite laminates

Sl. no.	Fe_2_O_3_ (wt%)	Number of layers	Epoxy : hardener (vol, %)	Sample designation
1	0	1	100 : 20	CF/E-1Layer
2		2		CF/E-2Layer
3		4		CF/E-4Layer
4	1	1		CF/1FE/E-1Layer
5		2		CF/1FE/E-2Layer
6		4		CF/1FE/E-4Layer
7	2	1		CF/2FE/E-1Layer
8		2		CF/2FE/E-2Layer
9		4		CF/2FE/E-4Layer
10	3	1		CF/3FE/E-1Layer
11		2		CF/3FE/E-2Layer
12		4		CF/3FE/E-4Layer

### 2.4. Characterization

The morphology of the Fe_2_O_3_ nanofiller was studied by scanning electron microscopy (SEM; CARL ZEISS, Germany). The crystalline structure of the Fe_2_O_3_ nanoparticles was identified using powder X-ray diffraction (XRD; Rigaku MiniFlex 600, Japan) which ran at scanning speed in a 2*θ* range of 20° to 80°. Fourier Transform Infrared spectroscopy (IR Spirit, Shimadzu, Japan) was performed on the composite and each of its components to capture any interactions. For mechanical characterization, the test samples were cut using an abrasive water jet machining as per ASTM standards. The samples for the tensile test were prepared according to ASTM D-638.^21^

#### 2.4.1. Electromagnetic interference shielding test

The shielding effectiveness of the composite laminates was evaluated using the coaxial transmission line method, wherein the scattering parameters (*S*-parameters: *S*_11_, *S*_12_, *S*_21_, and *S*_22_) were measured with a two-port Vector Network Analyzer (VNA), model Keysight Technologies N1991A MY58312077, integrated with a WR-90 waveguide specimen holder.

Among the measured parameters, *S*_11_ (reflection coefficient at port 1) quantifies the portion of incident power reflected back from the material, while *S*_21_ (transmission coefficient from port 1 to port 2) indicates the amount of electromagnetic power transmitted through the specimen. Parameters *S*_12_ and *S*_22_, typically associated with reverse transmission and reflection at port 2, were considered less critical for this application and thus excluded from the analysis. The *S*_21_ parameter is of primary importance in determining shielding effectiveness, as it directly correlates with the transmitted power and, therefore, the attenuation capability of the shielding material.

The specimens for EMI shielding (22.86 mm × 10.16 mm) were cut with an abrasive water jet machine from the prepared CF/E and CF/FE/E laminates with strict dimensional tolerances and high edge quality. The samples were mounted in a WR-90 rectangular waveguide sample holder, where the scattering parameters (*S*-parameters) were measured at frequencies between 8 and 12 GHz. Each specimen was inserted into the measurement system after performing a total two-port calibration and recording the *S*-parameters from a baseline for each specimen to minimise systematic error.

**Fig. 3:**
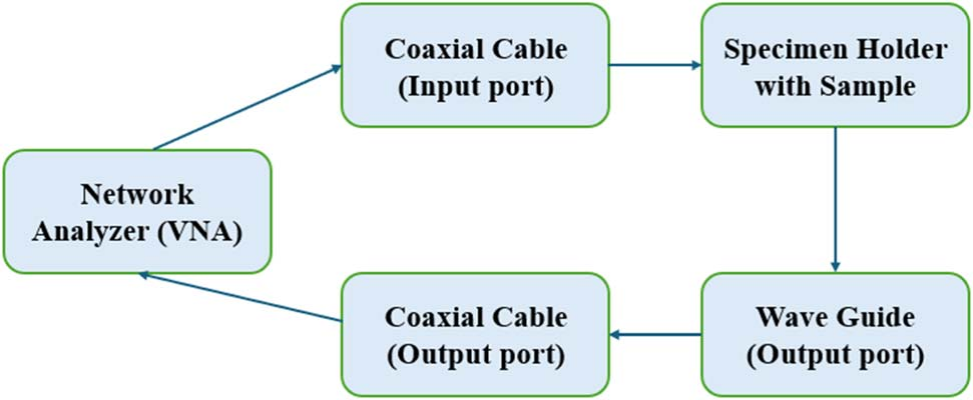
Experimental setup for measuring electromagnetic interference (EMI) shielding effectiveness.

As shown in Fig. 3, the block diagram depicts the experimental setup for evaluating EMI shielding effectiveness (SE) in the X-band frequency range (8.2–12.4 GHz) using a rectangular waveguide. The vector network analyzer (VNA) generates and receives microwave signals using two coaxial ports. The signals are generated on port 1 and transmitted *via* a coaxial cable into the waveguide input, where the test specimen is mounted in a custom holder. The attenuated or transmitted signals exit through the waveguide output and are then directed to port 2 of the VNA. In this setup, it is now possible to measure the scattering parameters (*S*_11_ and *S*_21_) to evaluate a specimen's shielding performance. Total shielding effectiveness was computed from the measured forward transmission as given in eqn (1) and (2) of the manuscript consistent with waveguide SE formulations reported for planar samples. Because the WR-90 fixture with a flat, isotropic specimen is a passive reciprocal two-port, *S*_21_ = *S*_12_ by reciprocity and the diagonal terms are dominated by the same port reflections; thus, reporting *S*_11_ and *S*_21_ suffices and is standard practice in SE studies using waveguides/VNAs. We now cite representative sources and general standards to support these choices.

All EMI shielding measurements were conducted in a controlled laboratory environment at 23 ± 2 °C and 50 ± 5% relative humidity, with specimens conditioned under the same conditions for 48 h prior to testing. The vector network analyzer (VNA) was calibrated using a thru-reflect-line (TRL) standard provided with the WR-90 waveguide kit to eliminate systematic errors from cables, connectors, and fixtures. Calibration was repeated before each measurement series to ensure stability. By reporting these environmental controls and calibration procedures, we provide greater transparency and confidence in the accuracy of the shielding effectiveness data.

The shielding effectiveness due to absorption (SE_A_) and reflection (SE_R_) was determined from the measured *S*-parameters using eqn (1) and (2), respectively:1
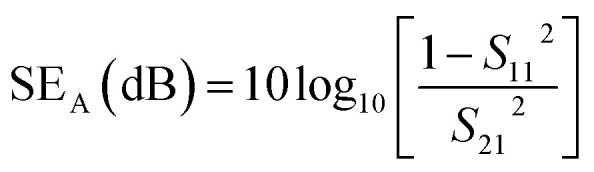
2
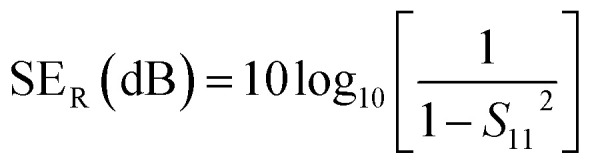


The total shielding effectiveness (SE_T_) was then computed by summing the contributions from absorption and reflection, as shown in eqn (3).

3 SE_T_ = SE_A_ + SE_R_

## 3. Results and discussion

### 3.1. Morphology of Fe_2_O_3_

**Fig. 4:**
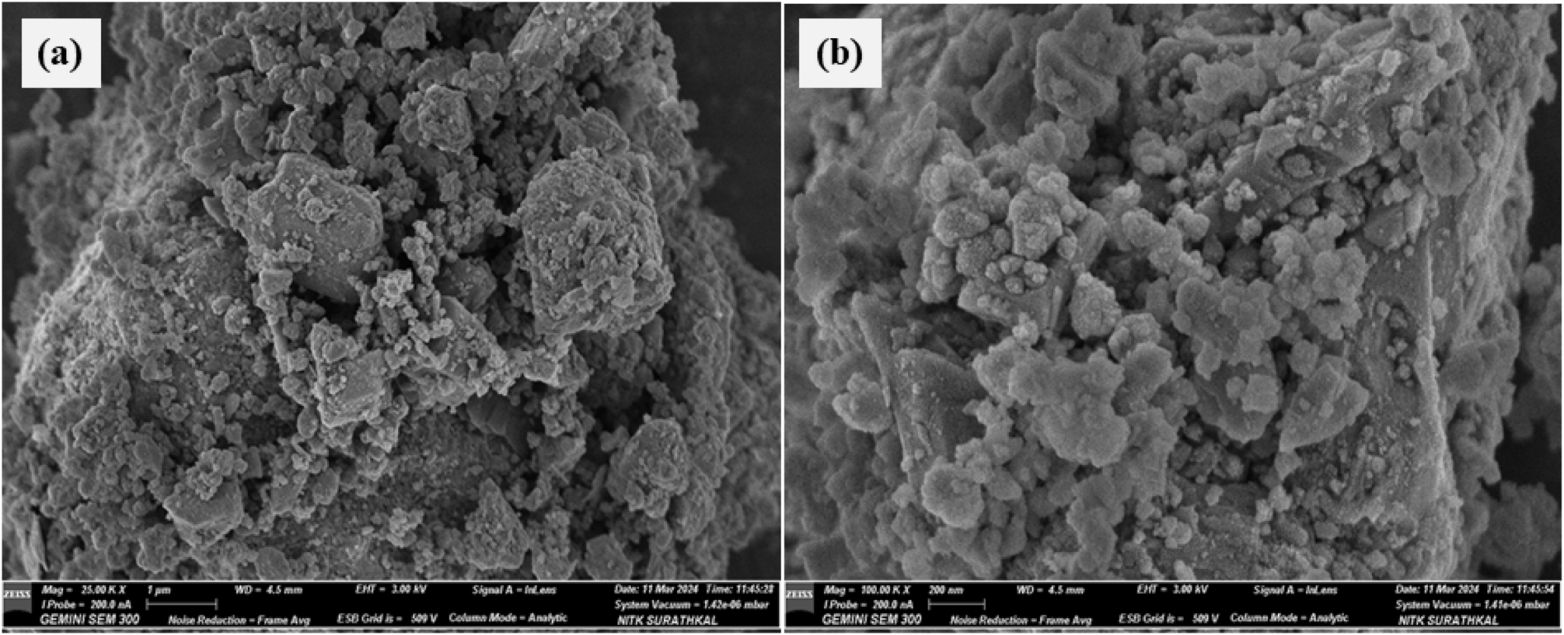
FESEM images of Fe_2_O_3_ nano filler at (a) 25 000× and (b) 100 000× magnification.

The morphology of the Fe_2_O_3_ nanofillers was investigated using SEM at various magnifications, and the obtained images are presented in Fig. 4. Fig. 4(a) shows the SEM image of Fe_2_O_3_ at 25 000× and Fig. 4(b) at 100 000× magnification. It is found that the Fe_2_O_3_ nanofillers used in this study are a mixture of spherical and irregular morphologies.

At 250 00× magnification (refer Fig. 4(a)), the Fe_2_O_3_ nanoparticles exhibit a highly agglomerated and irregular cluster-like structure. The particles appear to form dense aggregates, likely due to strong van der Waals forces and magnetic interactions characteristic of iron oxide nanoparticles. The surface is rough, with visible granularity indicating the polycrystalline nature of Fe_2_O_3_. At 100 000× magnification (refer Fig. 4(b)), the fine nanostructures within the agglomerates become clearer. The particles appear to have a roughly spherical to polyhedral shape, with an average size likely in the 20–80 nm range (based on visual approximation). The higher magnification reveals that these clusters are formed from much smaller primary nanoparticles.

The primary particle size (in Fig. 4(b)) seems to be in the nanometer range, but the secondary clusters (visible in Fig. 4(a)) are significantly larger, spanning micrometre scales. This hierarchical structure such as small primary nanoparticles forming larger agglomerates is typical for Fe_2_O_3_ due to high surface energy and magnetic dipole–dipole interactions. The particle distribution appears non-uniform, with some densely packed regions and others showing voids or gaps, which may affect dispersion if used as a filler in composites.

### 3.2. XRD analysis of Fe_2_O_3_

**Fig. 5:**
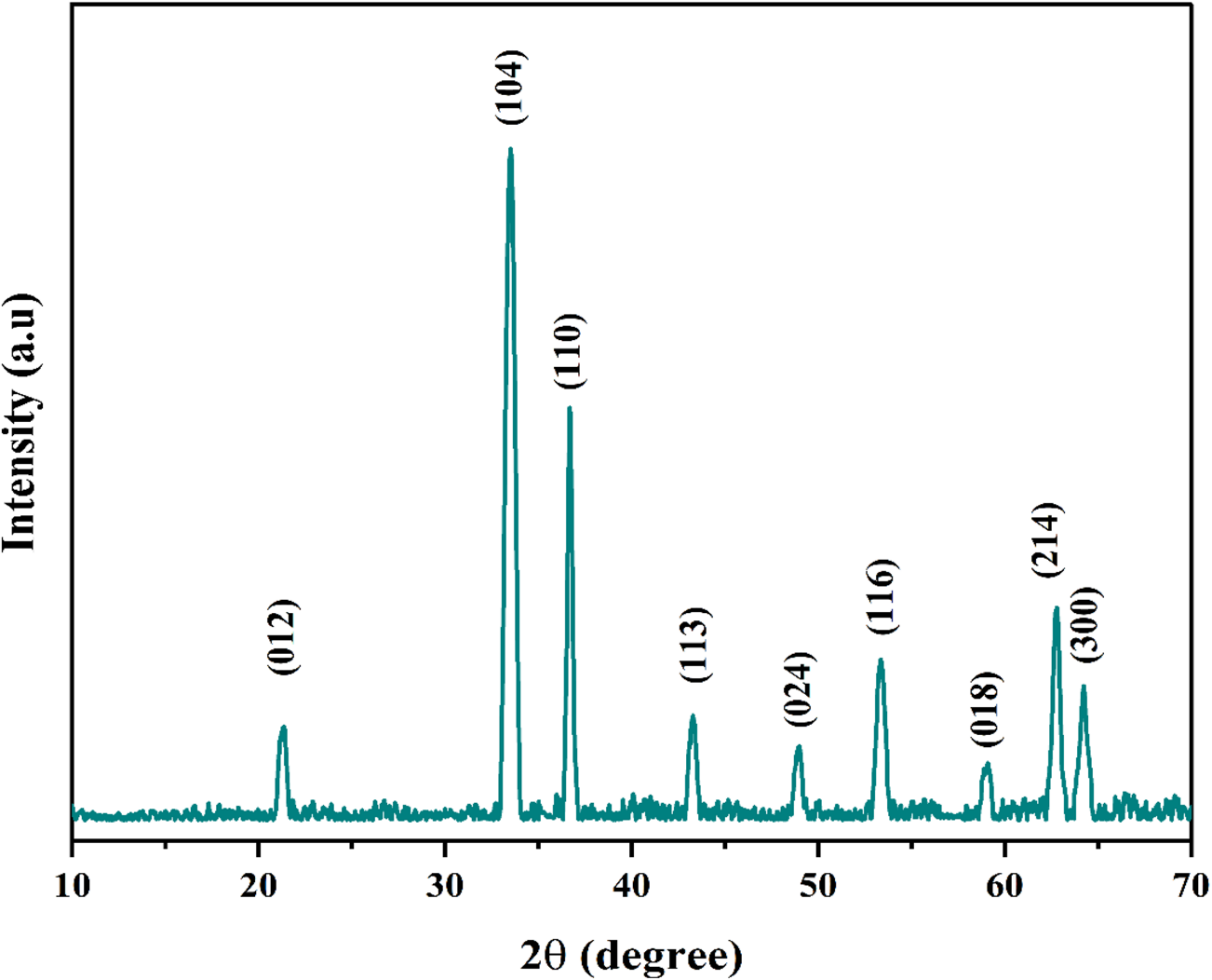
X-RD spectra of Fe_2_O_3_ nanoparticles.

Powder X-ray diffraction (XRD) was used to verify the phase and crystal structure of the Fe_2_O_3_ nanoparticles employed in this study. The XRD pattern of the Fe_2_O_3_ nanoparticles as received is shown in Fig. 5. The observed diffraction peaks at 21.2°, 33.4°, 36.7°, 43.3°, 49.04°, 53.3°, 59.14°, 62.7°, and 64.2°, correspond to the characteristic reflections of hematite with a hexagonal crystal system (space group *R*3̄*c*), with lattice parameters of *a* = *b* = 5.034 Å and *c* = 13.748 Å (JCPDS no. 033-0664). As no additional diffraction peaks were identified, this also shows that the Fe_2_O_3_ nanoparticles are of high purity, and this agrees with the stated 99% purity.

### 3.3. FTIR spectra

**Fig. 6:**
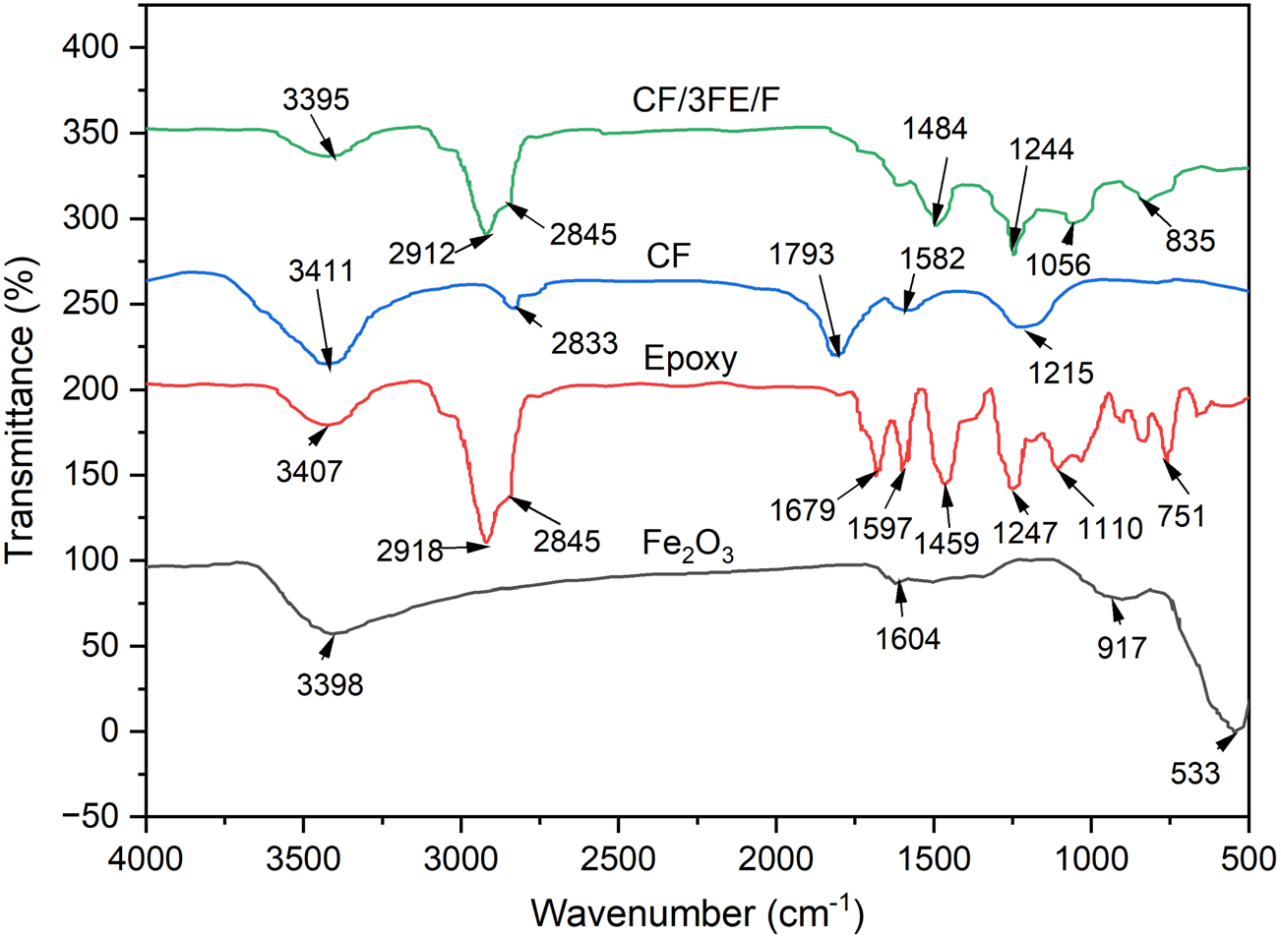
FTIR spectra of Fe_2_O_3_ nanoparticles, epoxy, carbon fabric and carbon fabric/Fe_2_O_3_/epoxy (CF/3FE/F) composite.

The FTIR spectrum of Fe_2_O_3_/carbon fabric/epoxy (CF/3FE/F) composite is compared with the FTIR spectra of the individual components Fe_2_O_3_, carbon fabric, and epoxy, respectively, as shown in Fig. 6. Table 3 presents the peak assignments, and the respective functional groups present in the samples. One can see that the FTIR spectrum of the CF/3FE/F composite reveals the successful incorporation of the peaks from all three constituents without any substantial chemical degradation or change in the functional groups. The IR spectrum of the composites incorporates peaks originating from CF and Fe_2_O_3_, such as O–H, C–H, and Fe–O vibrations, indicating successful integration of the reinforcements in the composite. The absence of any significant peak shifts or new, strong bands indicates that the interaction of the constituents in the composite is mainly due to the physical interaction. However, the minor broadening and intensity variations at certain regions (*e.g.*, 3380–3400 cm^−1^ and 1200–1500 cm^−1^) imply the presence of interfacial hydrogen bonding and potential interactions between Fe_2_O_3_ and the polymer matrix. These interactions between the epoxy-Fe_2_O_3_ can strengthen the epoxy-CF adhesion and can expect an enhancement in the mechanical properties as compared to composites without Fe_2_O_3_ nanoparticles.

**Table 3:** FTIR peak assignments and functional group analysis

Wavenumber (cm^−1^)	Functional group/vibrationation mode	Origin
3400	O–H stretching	Fe_2_O_3_, CF, epoxy, composite
2912, 2845	C–H stretching	CF, epoxy, composite
1793	C=O stretching	CF
1679–1604	O–H bending/aromatic	Fe_2_O_3_, epoxy
1582	C=C stretching	CF, composite
1484–1459	Aromatic C–H, ring	Epoxy, composite
1247–1244, 1215	C–O–C stretching (epoxy)	Epoxy, composite
1110–1056	C–O stretching	Epoxy, composite
917, 835	Fe–O vibration/aromatic ring	Fe_2_O_3_, composite
751–533	Fe–O vibration/aromatic	Fe_2_O_3_, epoxy, composite

The observed bands in the 470–580 cm^−1^ region corroborate Fe–O vibrations of α-Fe_2_O_3_, distinguishing physical adsorption from chemical bonding at the polymer–filler interface requires thermal or surface-energetic evidence. We therefore now frame the FTIR discussion as indicative rather than conclusive and note that DSC/TGA and contact-angle/XPS measurements would be appropriate to substantiate interfacial mechanisms in future work.

### 3.4. Tensile behaviour of composites

**Fig. 7:**
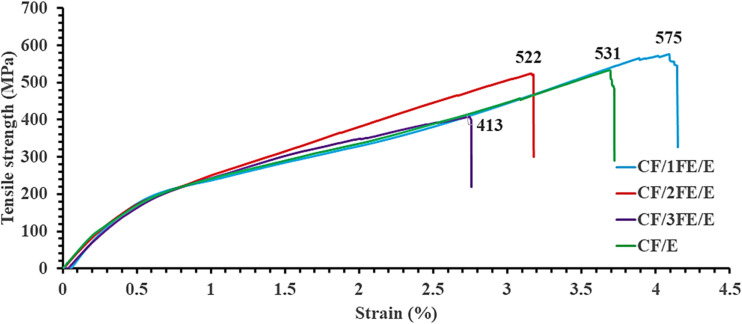
Tensile behaviour of carbon fabric and Fe_2_O_3_ reinforced epoxy (CF/FE/E) composites.

In this previous study, we presented tensile behaviour of pristine epoxy, which was reinforced with bi-directional carbon fabric composite (CF/E) and value as the control material. These results are presented here in relation to those of epoxy carbon fabric composites with 1, 2, or 3 wt% Fe_2_O_3_ (CF/1FE/E, CF/2FE/E and CF/3FE/E), which are displayed in Fig. 7. The concentration of the loaded Fe_2_O_3_ nanoparticles substantially influences the mechanical properties of the composites. At low concentrations of Fe_2_O_3_ (1 wt%), tensile properties of the composite improved due to better interfacial bonding between the carbon fibres and epoxy matrix. When interfacial bonding strength improves, load transfer is improved and early failure of composites is delayed through the mechanism predominantly through interfacial hydrogen bonding as evidenced by FTIR. Nanoparticles also assist in uniform stress distribution within the polymer matrix that can potentially restrict crack initiation and propagation. At higher concentrations (3 wt%), filler agglomeration leads to a decline the tensile strength. Sun *et al.* reported that 1 wt% of Fe_2_O_3_ nanoparticles yielded the best tensile properties in carbon fabric/epoxy composites, whereas higher concentrations resulted in diminished mechanical performance due to poor dispersion.^22^ The present study shows a similar trend. Overall, adding Fe_2_O_3_ nanoparticles to carbon fabric epoxy composites generally increases tensile strength and modulus, with optimal results around 1–2 wt% nanoparticle loading. However, strain-to-failure and tensile strength slightly decrease at loadings above 2 wt% because of reduced ductility and agglomeration. At 3 wt% Fe_2_O_3_, agglomeration may occur, reducing reinforcement effectiveness and decreasing the composite's tensile properties.

Neat epoxy is highly flaw-sensitive under tensile loading, small variations in cure, micro voids, or surface defects act as critical flaws that trigger failure, producing wide specimen-to-specimen scatter. Also, processing related microstructure often differs between neat and filled systems that directly affect tensile strengths. Small amounts of nanoparticles can reduce effective void content by occupying free volume will increase if dispersion is poor. In these cases, both voids and partial cure reduce tensile strength and increase test-to-test variability. Similarly, well dispersed nanoparticles can stabilize the microstructure and narrow the scatter. Fe_2_O_3_ additions can create competing mechanisms that make the filled curves look “trendless” unless dispersion is tightly controlled. At very low loadings, well-dispersed Fe_2_O_3_ can increase tensile strength *via* crack deflection/pinning and improved interfacial polarization-induced energy dissipation, at higher loadings, agglomerates behave as stress concentrators that depress strength and re-introduce scatter. Therefore, the trend depends upon the balance between dispersion-assisted toughening and agglomeration-induced embrittlement.

**Fig. 8:**
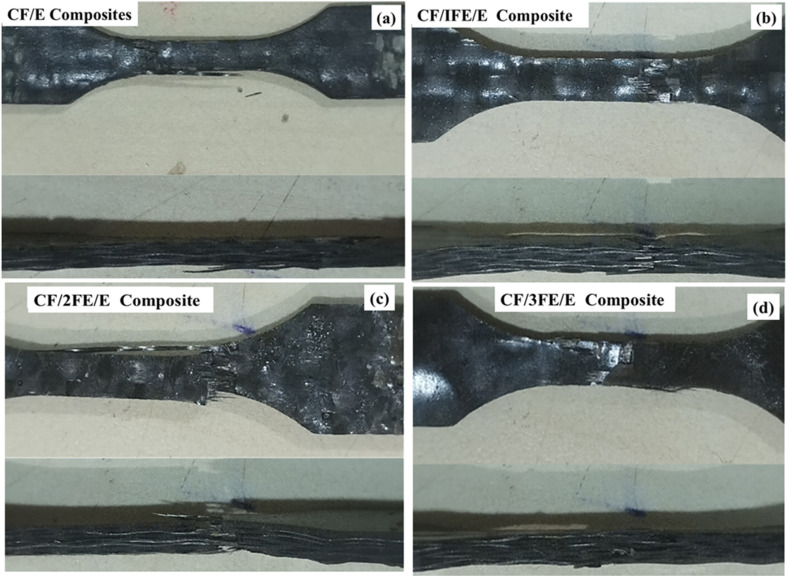
Fractured tensile specimen of (a) CF/E composite, (b) CF/1FE/E composite, (c) CF/2FE/E composite, (d) CF/3FE/E composite.

Referring to the tensile failure sample of CF/E composites previously reported by our group, which is shown in Fig. 8(a),^23^ the tensile failure of CF/FE/E composite samples in this study is displayed in Fig. 8(b), (c), and (d). It is observed that the CF/E and CF/FE/E samples exhibited similar behaviour, with less delamination and good integrity of filler, fibre, and matrix, as shown in Fig. 8(a) and (b). The sample with 2 wt% Fe_2_O_3_ filler showed matrix cracking with delamination, as shown in Fig. 8(c), and this failure was more severe in the 3 wt% filler-loaded sample, as shown in Fig. 8(d). This indicates that at higher Fe_2_O_3_ concentrations, the material failed due to brittle fracture of the matrix and increased delamination. In the current study, the failure analysis presented in Fig. 8 is qualitative, showing features such as river markings, fibre pull-out, and matrix cracking that are indicative of interfacial adhesion and filler–matrix interactions. While this provides useful insight, we acknowledge the limitation that no quantitative fractographic metrics (*e.g.*, crack propagation length, void density, or fibre-matrix debonding area) were extracted.

**Fig. 9:**
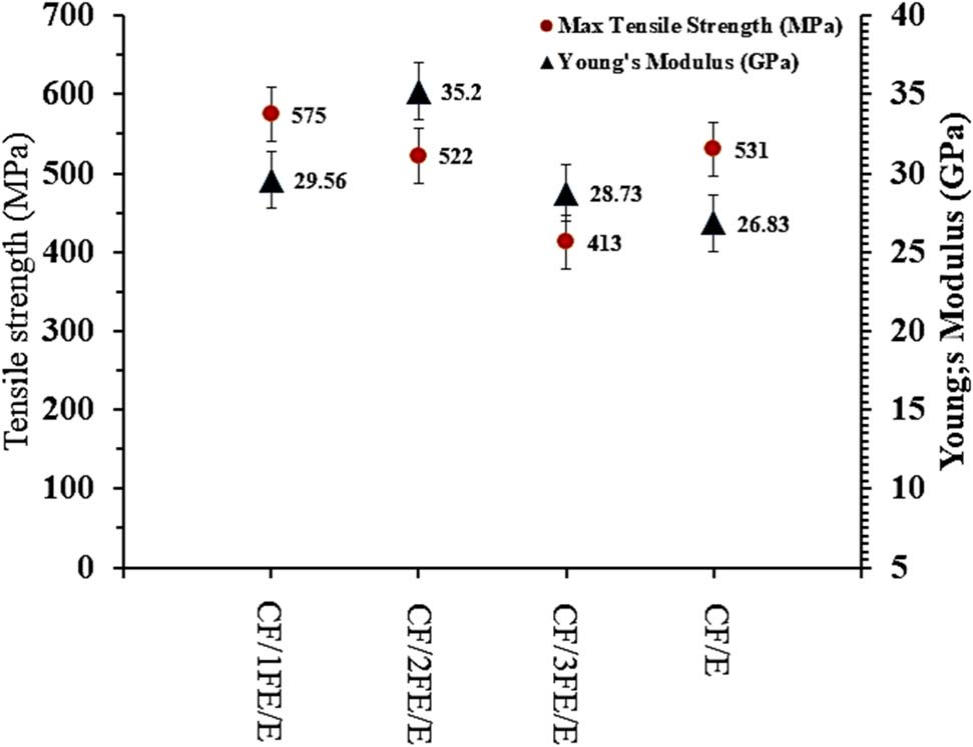
Comparison of tensile strength and Young's modulus of CF/E with CF/FE/E composites.

From the tensile stress *versus* strain plot, the Young's modulus of the neat CF/E composite is computed and found to be 26.83 GPa and a maximum tensile strength of 531 MPa. Incorporation of Fe_2_O_3_ significantly influenced stiffness, with a maximum modulus of 35.20 GPa observed for CF/2FE/E, representing a ∼31% increase over the neat composite shown in Fig. 9. This enhancement is attributed to improved stress transfer due to strong interfacial adhesion between the Fe_2_O_3_-modified epoxy matrix and the CF reinforcement, consistent with findings by Shukla (2019) and Ahmad *et al.* (2021), where optimal nanofiller content improved modulus *via* synergistic reinforcement mechanisms.^4,6^ In terms of tensile strength, the highest value (575 MPa) was recorded for CF/1FE/E, marginally surpassing the neat composite. However, strength declined at higher loadings, with CF/3FE/E showing the lowest value (413 MPa). This reduction is attributed to nanoparticle agglomeration, void formation, and reduced fibre–matrix interfacial integrity at excessive filler content, as similarly reported in studies on nanoparticle-reinforced polymer composites.^8,24^ Overall, the results indicate that moderate filler addition (1–2 wt%) optimizes stiffness, while excessive loading compromises tensile strength despite maintaining high modulus. This trend aligns with literature highlighting the balance between dispersion quality, interfacial bonding, and filler content in determining mechanical performance.^24,25^

## 4. Electromagnetic shielding effectiveness

**Fig. 10:**
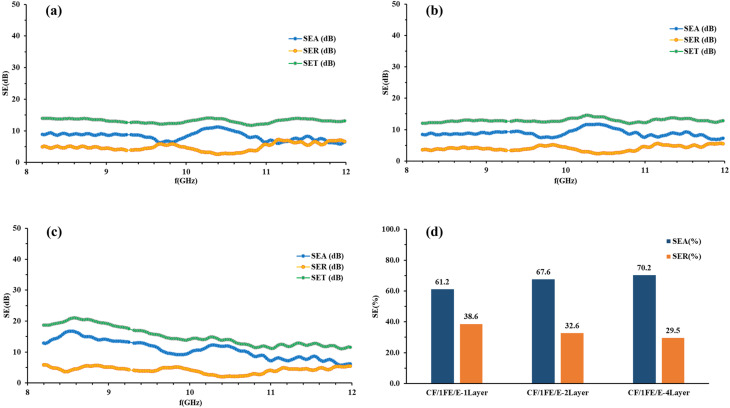
SE_A_, SE_R_, and SE_T_ of (a) CF/1FE/E single-layer, (b) CF/1FE/E double-layer, (c) CF/1FE/E four-layer composites, and (d) the corresponding SE_A_ and SE_R_ percentage contributions for all CF/1FE/E samples.

**Fig. 11:**
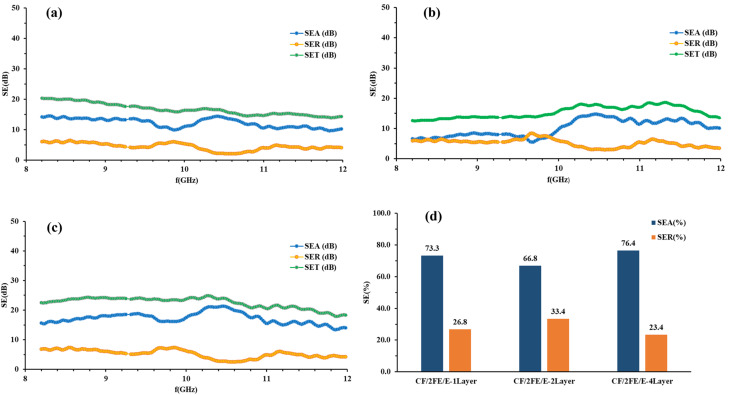
SE_A_, SE_R_, and SE_T_ of (a) CF/2FE/E single-layer, (b) CF/2FE/E double-layer, (c) CF/2FE/E four-layer composites, and (d) the corresponding SE_A_ and SE_R_ percentage contributions for all CF/2FE/E samples.

**Fig. 12:**
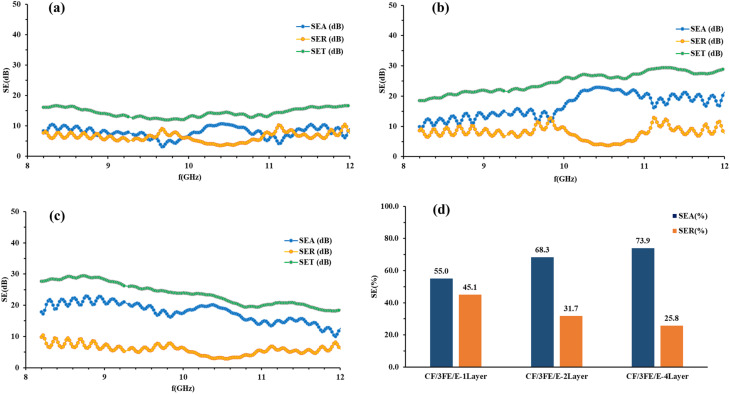
SE_A_, SE_R_, and SE_T_ of (a) CF/3FE/E single-layer, (b) CF/3FE/E double-layer, (c) CF/3FE/E four-layer composites, and (d) the corresponding SE_A_ and SE_R_ percentage contributions for all CF/3FE/E samples.

The electromagnetic interference shielding effectiveness (EMI SE) of a material represents its capacity to attenuate or block incident electromagnetic waves. The calculated values of SE_A_, SE_R_ and SE_T_ for CF/1FE/E, CF/2FE/E, and CF/3FE/E composite laminates in single-layer, double-layer, and four-layer configurations were plotted over the 8–12 GHz frequency range. The corresponding results are presented in Fig. 10(a–c), 11(a–c), and 12(a–c), respectively.

From previous studies on CF/E composites in the X-band, we determined that the SE_A_ of the single- and double-layer samples remained relatively stable throughout the frequency range, while the four-layer samples exhibited serious frequency-dependent variability in SE_A_ due to the skin effect. The skin depth, defined as the extent to which an electromagnetic wave will penetrate a material, is inversely proportional to the square root of frequency (*f*) which is displayed in (4). Note that *δ* represents the skin depth, *μ* is magnetic permeability (H m^−1^), *σ* is electrical conductivity (S m^−1^), and *f* is the frequency of the impinging electromagnetic field.4
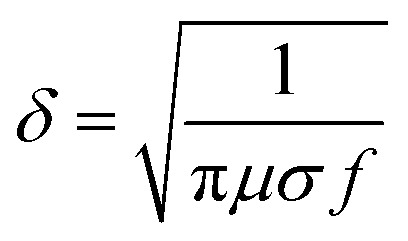


According to eqn (4), as the skin depth *δ* decreases as frequency increases, means that, at higher frequencies, EM waves tend to travel along the surface rather than penetrate the material. At lower frequencies, larger *δ* allows for deeper penetration and greater absorption, which explains the superior low-frequency performance of four-layer samples. Increasing the number of layers enhances absorption-dominated shielding, as multiple carbon fabric layers better attenuate EM waves through successive absorption and reduced reflection.

Fig. 10(a–c) presents the SE_A_, SE_R_, and SE_T_ of CF/1FE/E composites with single-, double-, and four-layer structures in the X-band region. As depicted in Fig. 10(d), the SE_A_ contribution increases from 61.2% to 67.6% with the transition from a single to a two-layer configuration. A comparable trend in SE_A_ and SE_R_ is observed for the two- and four-layer samples. Notably, the SE_R_ of the CF/1FE/E composite is lower than that of the CF/E system, which can be attributed to the enhanced absorption of EM radiation by the Fe_2_O_3_ fillers. Therefore, incorporation of Fe_2_O_3_ promotes absorption-dominated shielding effectiveness in the composite.

SE_A_, SE_R_, and SE_T_ of CF/2GNP/E composites with one-, two-, and four-layer structures in the X-band are shown in Fig. 11(a–c), and the percentage SE is illustrated in Fig. 11(d).

CF/2FE/E composites demonstrate absorption-dominated EMI shielding in all configurations, with SE_T_ and SE_A_ values increasing as the number of layers increases. The four-layer laminate achieves the highest shielding effectiveness (∼22 dB), confirming the benefit of layer stacking in enhancing performance. SE_A_ contributes more than 66% to the overall shielding across all samples, indicating that absorption is the primary shielding mechanism in the X-band region.

In the X-band region, the SE_A_, SE_R_, and SE_T_ values of the CF/3FE/E composites with single, double-, and four-layer configurations are represented in Fig. 12(a–c) and the corresponding percentage SE is given in Fig. 12(d). The presence of 3 wt% Fe_2_O_3_ as a filler increased the absorption-related shielding effectiveness due to a more efficient conductive network formed in the epoxy; in addition, multilayered structures also augmented absorption-dominated shielding. The two-layer CF/3FE/E demonstrably the best overall shielding effectiveness at 25.3 dB among the tested samples, which indicates significant potential for practical use in the X-band frequency range.

The shielding effectiveness and mechanisms in case of GNP-based epoxy composites is that graphene nanoplatelets contribute primarily through high electrical conductivity and the formation of percolated conductive networks. In this case the dominant shielding mechanism is reflection due to impedance mismatch and is supplemented by some absorption *via* multiple scattering inside the conductive network. So, these systems achieve relatively high SE values at moderate loadings. In case of carbon fabric with Fe_2_O_3_ nanoparticle–epoxy composites, carbon fibers provide a long range continuous conductive pathways and mechanical reinforcement. Also, Fe_2_O_3_ nanoparticles introduce magnetic loss (natural resonance, eddy current loss) and enhance interfacial polarization. Thus, shielding arises from a synergistic combination of reflection from conductive carbon fabric, absorption from Fe_2_O_3_-induced magnetic/dielectric losses, and multiple scattering due to heterogeneous interfaces. So, compared with GNP alone, this dual-action usually yields higher absorption-dominated shielding, which is desirable in applications where weight reduction, structural integrity and EMI performance must be simultaneously achieved.

**Fig. 13:**
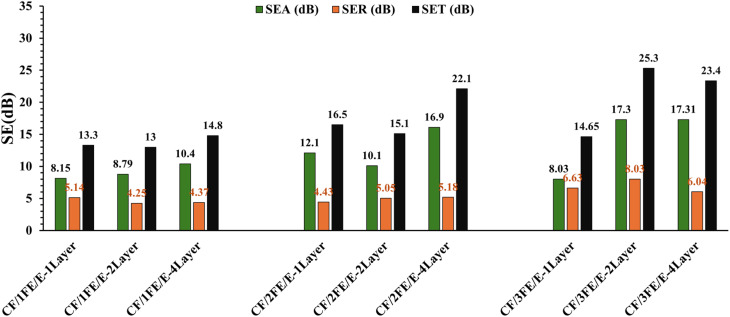
SE_A_, SE_R_ and SE_T_ of CF/FE/E composites.

Fig. 13 illustrates the comparative analysis of CF/1FE/E, CF/2FE/E, and CF/3FE/E composites across single-, double-, and four-layer configurations, revealing a clear trend of increasing total shielding effectiveness (SE_T_) with higher filler content and additional layers. Among all, CF/3FE/E-2Layer exhibits the maximum SE_T_ of 25.3 dB, closely followed by CF/3FE/E-4Layer (23.4 dB). SE_A_ consistently dominates over SE_R_ in all samples, indicating absorption as the primary EMI shielding mechanism, enhanced by Fe_2_O_3_-induced magnetic losses and carbon fabric conductivity. Stacking layers boosts SE_A_*via* repeated internal reflections; however, the higher Fe_2_O_3_ content strengthens dielectric and magnetic losses, resulting in excellent X-band shielding performance. In aerospace and defence sectors, EMI shielding materials are generally expected to achieve ≥30 dB attenuation, which corresponds to ∼99.9% reduction of incident electromagnetic radiation across the relevant frequency ranges (typically 8–12 GHz or even broader). For critical avionics enclosures or military-grade applications, the requirement is even stricter, often ≥60–80 dB to protect sensitive electronic systems and ensure electromagnetic compatibility (EMC). Civil aircraft structural composites are usually benchmarked against the 30 dB threshold, balancing performance with weight reduction and cost. The 25.3 dB SE_T_ value reported here corresponds to ∼99.7% attenuation. While this is a strong result for a lab-developed epoxy composite with Fe_2_O_3_ nanoparticles and carbon fabric, it falls slightly below the minimum 30 dB level generally recommended for aerospace-grade shielding panels. This study achieves near-threshold performance with a structural composite (carbon fabric reinforced epoxy + Fe_2_O_3_) is significant because it demonstrates dual functionality of mechanical integrity and EMI shielding which is a crucial step toward lightweight multifunctional aerospace materials. Table 4 compares the EMI-SE of various carbon fabric composites reported previously and in the present study. The present study reports a maximum SE of 25.3 dB, this value should be interpreted relative to filler loading, laminate thickness, and electrical conductivity. Unlike several literature examples where high SE values (>50 dB) are achieved at substantially higher filler concentrations (*e.g.*, 40–60 wt% Fe_3_O_4_, CNT/graphite systems, or dual nanofillers) and thicker laminates (>20–100 layers), our design intentionally maintains low filler content (1–4 wt% Fe_2_O_3_) and relatively thin laminates (1–3 layers, ∼2–3 mm thickness) to balance mechanical integrity with processability. Consequently, the lower conductivity of our composites compared to highly conductive matrices (*e.g.*, MXene- or CNT-based systems) inherently limits SE, as predicted by classical shielding theory where SE ∝ *σ*^0.5^ × *t*. However, the present study demonstrates that even with minimal filler loading and simpler hand lay-up processing, SE values in the 13–25 dB range are achievable, which is significant for lightweight EMI shielding applications. Furthermore, our results are consistent with other low-filler, low-thickness Fe_2_O_3_-based systems, which typically yield SE values in the 20–30 dB range, underscoring the material's comparative efficiency.

**Table 4:** Comparison of the EMI SE of various carbon fabric composites from previous studies and the present work

Matrix, primary reinforcement & secondary reinforcement			Process	*N*	*t* (mm)	*σ* (S cm^−1^)	SE			Ref.
Fibre/fabric/matrix	Filler	Concentration (wt%)					SE_A_ (dB)	SE_R_ (dB)	SE_T_ (dB)	
T800-CF	MWCNT + Fe_2_O_3_	10	Prepreg + compression molding	20	3.0	35.0	∼50	∼10	59.0	6
C fabric-plain weave	Fe_3_O_4_	60	Solution casting + hot pressing	1	1.54	1.17	∼47.1	∼3.8	∼58.93	7
PTFE + CNT dual NF	Fe_3_O_4_ + MXene	50 + 70	Shear-induced *in situ* fibrillation + vacuum filtration	2	0.0849	4.0278	∼24.8	∼19.8	44.56	8
CFAN-artificial nacre	Fe_2_O_3_	<10	Matrix-directed mineralisation + lamination	∼100	<0.001	—	∼21–25	∼4–7	∼27–29	9
rGCE-aerogel	Fe_3_O_4_	10	*In situ* chemical precipitation	—	2.5	—	—	—	−58.13	10
TPU	Graphite + CoFe_2_O_4_	40 + 40	Melt mixing + compression molding	3 (G/F/G)	2.4	—	—	—	54	11
PA11/PA + SCF	—	30	Extrusion + compression molding	1	1	∼2.28 × 10^−5^	∼22.7	∼5.3	28	12
UDCF/E prepreg (T800, 12 K)	Gp + Fe_2_O_3_	4 + 6	Hand lay-up followed by compression molding)	16 [0/90]_8_	2.5	G-4	∼90.5	∼5	95.57	13
						F-6				
C fabric/E	GO + Fe_3_O_4_	8	Layer-by-layer deposition + hot press curing	8	∼1.35	∼3.6	22.8	10.7	32.9	14
Epoxy resin	Fe_3_O_4_ + CB	15 + 50	Mixing + casting	—	0.7	—	11.85	−17.6	36.6	15
Epoxy resin	GNP + Fe_3_O_4_	12 + 4	Melt blending	1	1	—	—	—	∼7	16
	Fe_3_O_4_ decorated GNP	12 + 4							9	
Cotton fibre/epoxy	Fe_3_O_4_ + GNP	5 + 30	Epoxy impregnation + hot press	2	2.68	—	∼28.1	∼5.0	33.1	17
C fabric-plain weave			Hand lay-up method	1			7.8	6.0	13.9	23
				2			10.2	8.8	19	
				4			12.6	8.0	20.6	
Present study	Fe_2_O_3_/CF	1	Hand lay-up method	1		—	8.15	5.14	13.3	
				2			8.79	4.25	13	
				4			10.4	4.37	14.8	
		2		1		—	12.1	4.43	16.5	
				2			10.1	5.05	15.1	
				4			16.9	5.18	22.1	
		3		1		—	8.03	6.63	14.6	
				2			17.3	8.03	25.3	
				4			17.3	6.04	23.4	

The improvement in tensile strength observed at moderate Fe_2_O_3_ loadings reflects effective stress transfer at the fiber–matrix interface, which simultaneously promotes the formation of continuous conductive or semi-conductive networks essential for EMI attenuation. Conversely, the reduction in mechanical properties at higher filler contents can be attributed to agglomeration and interfacial defects, which not only act as stress concentrators but also disrupt the uniformity of the shielding pathways, thereby limiting shielding effectiveness despite increased filler volume. Quantitatively, this trade-off can be rationalized by considering that both load transfer efficiency (*σ* ∝ *V*_f_ × *τ*_i_, where *τ*_i_ is interfacial shear strength) and shielding effectiveness (SE ∝ *σ*^0.5^ × *t*, where *σ* is conductivity and *t* is thickness) are maximized under conditions of uniform dispersion and strong interfacial bonding. By correlating tensile strength retention with SE trends across different filler loadings, we demonstrate that optimal performance arises not simply from increasing filler concentration, but from achieving synergistic dispersion and interfacial compatibility. This mechanistic perspective situates our findings within broader composite design principles and highlights pathways for future optimization (*e.g.*, surface functionalization of Fe_2_O_3_, hybrid filler strategies, or controlled laminate stacking) to simultaneously advance mechanical robustness and EMI shielding efficiency.

## 5. Conclusions

This work established that bidirectional carbon fabric/epoxy composites reinforced with Fe_2_O_3_ nanoparticles can simultaneously deliver mechanical robustness and effective electromagnetic shielding in the X-band frequency range. Optimal filler loading of 1–2 wt% improved tensile strength and stiffness through enhanced fiber–matrix interfacial bonding, while higher loading led to agglomeration and reduced strength. All composites exhibited absorption-dominated EMI shielding, with a maximum SE_T_ of 25.3 dB achieved in a two-layer laminate containing 3 wt% Fe_2_O_3_. The findings demonstrate that careful control of nanofiller concentration and laminate configuration enables the design of lightweight, multifunctional composites suitable for aerospace applications requiring structural integrity and electromagnetic compatibility.

The carbon fabric/Fe_2_O_3_ nanoparticle/epoxy (CF/3FE/E-Layer) composite developed in this study shows multifunctional characteristics as good mechanical integrity from carbon fabric, magnetic and dielectric loss from Fe_2_O_3_, and structural reliability from epoxy. While the aerospace sector is an obvious target, several other industries could benefit from such composites. Which included automotive and electric vehicles (EV) with increasing use of sensors, radar, and advanced driver-assistance systems (ADAS), EMI shielding is crucial for safety and reliability. Also, the composites can replace heavier metallic shielding, lower vehicle weight and improve fuel efficiency. These composites can also be used in EMI-safe enclosures for EV batteries and control units, reducing electromagnetic crosstalk. The carbon fabric/Fe_2_O_3_ nanoparticle/epoxy (CF/3FE/E-Layer) composite developed in this study also can be used in structural panels for antenna housings, base stations, and smart towers where both strength and EMI shielding are needed. Also, it can be used in laptops, tablets, and high-frequency devices that benefit from lightweight shielding to prevent interference.

To further improve the shielding effectiveness (SE_T_) beyond the current 25.3 dB, future work should focus on multilayer or gradient architectures that enhance multiple reflections, hybridization of Fe_2_O_3_ with conductive 2D fillers such as graphene or MXenes to combine magnetic and conductive losses, and surface functionalization of Fe_2_O_3_ to improve dispersion and interfacial polarization. In addition, thickness optimization guided by skin-depth calculations and the introduction of porous or foam-like sublayers can significantly increase absorption without adding excessive weight. Together, these strategies are expected to raise SE_T_ above the aerospace benchmark of 30 dB and broaden the applicability of the composites to advanced structural and electronic shielding applications.

## Conflicts of interest

There are no conflicts of interest to declare.

## Data Availability

Data supporting the results reported in this manuscript are included in this article.
